# Musical Imagery Involves Wernicke’s Area in Bilateral and Anti-Correlated Network Interactions in Musicians

**DOI:** 10.1038/s41598-017-17178-4

**Published:** 2017-12-06

**Authors:** Yizhen Zhang, Gang Chen, Haiguang Wen, Kun-Han Lu, Zhongming Liu

**Affiliations:** 1 Weldon School of Biomedical Engineering, Purdue University, West Lafayette, IN USA; 2 School of Electrical and Computer Engineering, Purdue University, West Lafayette, IN USA; 3 Purdue Institute for Integrative Neuroscience, Purdue University, West Lafayette, IN USA; 4 Scientific and Statistical Computing Core, National Institute of Mental Health, National Institutes of Health, Bethesda, MD USA

## Abstract

Musical imagery is the human experience of imagining music without actually hearing it. The neural basis of this mental ability is unclear, especially for musicians capable of engaging in accurate and vivid musical imagery. Here, we created a visualization of an 8-minute symphony as a silent movie and used it as real-time cue for musicians to continuously imagine the music for repeated and synchronized sessions during functional magnetic resonance imaging (fMRI). The activations and networks evoked by musical imagery were compared with those elicited by the subjects directly listening to the same music. Musical imagery and musical perception resulted in overlapping activations at the anterolateral belt and Wernicke’s area, where the responses were correlated with the auditory features of the music. Whereas Wernicke’s area interacted within the intrinsic auditory network during musical perception, it was involved in much more complex networks during musical imagery, showing positive correlations with the dorsal attention network and the motor-control network and negative correlations with the default-mode network. Our results highlight the important role of Wernicke’s area in forming vivid musical imagery through bilateral and anti-correlated network interactions, challenging the conventional view of segregated and lateralized processing of music versus language.

## Introduction

Mental imagery, including the experiences such as “seeing in the mind’s eye”, or “hearing in the head”, is a hallmark example of human cognitive abilities. It refers to quasi-perceptual mentation that resembles vivid perception in the absence of any external stimuli^1^. Previous studies suggest that imagery and perception tasks involve overlapping cortical areas^2–4^, which however may not code the same information^5^ or engage in the same network interactions^6^ during both tasks. To date, the neural coding and network bases of mental imagery remains largely unknown and has been a long-standing topic of active research^7,8^.

Among various types of mental imagery, musical imagery may occur with surprisingly high accuracy^9–11^. A famous example illustrating human capabilities in this arena was Ludwig van Beethoven; despite deafness in his later years, he continued composing and conducting great symphonies guided by his “inner ear”^12^. Neurobiologically, functional neuroimaging studies suggest that musical imagery involves cortical areas within and beyond the auditory system^13^, including the auditory belt areas^3,10,14,15^, the association cortex^15,16^, the prefrontal cortex^3,14,17^, and the premotor and supplementary motor areas^3,16–18^. Furthermore, it has become well established that such areas do not function in isolation, but interact in networks. In fact, the coordinated network interactions among these areas have been thought to underlie various aspects of musical imagery, e.g. auditory processing^3,15^, sensorimotor coordination^3,16,18^, memory retrieval^3,16^, cognitive control^10,18^, and emotion^3,18^. Hence, musical imagery is a rich behavioral and cognitive context, for which characterizing the patterns and dynamics of cortical networks may help understand the interplay of seemingly segregated functional systems.

To probe network interactions, it is necessary to gather sufficient samples of neural responses during musical imagery so that the functional relationships among different regions can be reliably measured. To meet this need, a musical-imagery task ought to be complex and sustained for several minutes or longer, especially if brain activity is observed with functional magnetic resonance imaging (fMRI). However, designing an experiment with musical imagery for such a prolonged period is challenging, because imagery is subjective, internally-driven, and thus difficult to control or access. For this reason, prior studies have focused on simple and short imagery tasks^3,15,19^ while treating the musical-imagery experience as a series of discrete events divided by periods of rest rather than as a continuous process. Although such studies have been useful for revealing activations with simple musical imagery, findings obtained from them cannot be used to conclusively state how activated regions interact to form functional networks or what information their responses represent during complex musical imagery.

Nonetheless, these limitations can be mitigated by using visual stimulation to guide musical imagery. Herholz *et al*. has used a karaoke-like visual display to cue subjects to imagine familiar songs for several seconds^3^. Although this methodology has not been used for a longer period of imagery, adopting a similar idea to control sustained musical imagery by using music visualization^20^ without lyrics may further dissociate music from language during imagery. Music visualization may enable musicians to vividly and repeatedly imagine a very complex music piece, such as a Beethoven symphony capable of arousing rich and dynamic emotion^21^. Moreover, repeated measures of sustained musical imagery allow separation of imagery-evoked responses from ongoing activity, making it possible to disentangle the task-evoked functional networks and spontaneously emerging (i.e. task-unrelated) networks^22,23^.

In this study, we aimed to map and compare both cortical activations and functional networks during musical imagery versus perception. Unlike prior studies, we used a silent music visualization to guide experienced musicians to repeatedly imagine an 8-minute symphony in the absence of external auditory input. The visualization served to inform subjects of the music content and control the timing for the subjects’ imagery experience, to maximize subjects’ ability to imagine the music in a similar way as they listened to it. The fMRI data acquired with this paradigm were evaluated for intra-subject reproducibility and functional connectivity^22,23^, to map cortical activations and functional networks under the sustained musical imagery paradigm or during the perception of musical stimulus. Moreover, we explored the information coded in the responses evoked by musical imagery and perception by correlating the fMRI signal with the time series of the auditory features of the music. Lastly, we compared imagery and perception-evoked functional networks with those in the resting state to explore the likely different patterns of network interactions during internally versus externally-driven mental processes.

## Results

### Visually-cued musical imagery evoked wide-spread cortical activation

**Figure 1:**
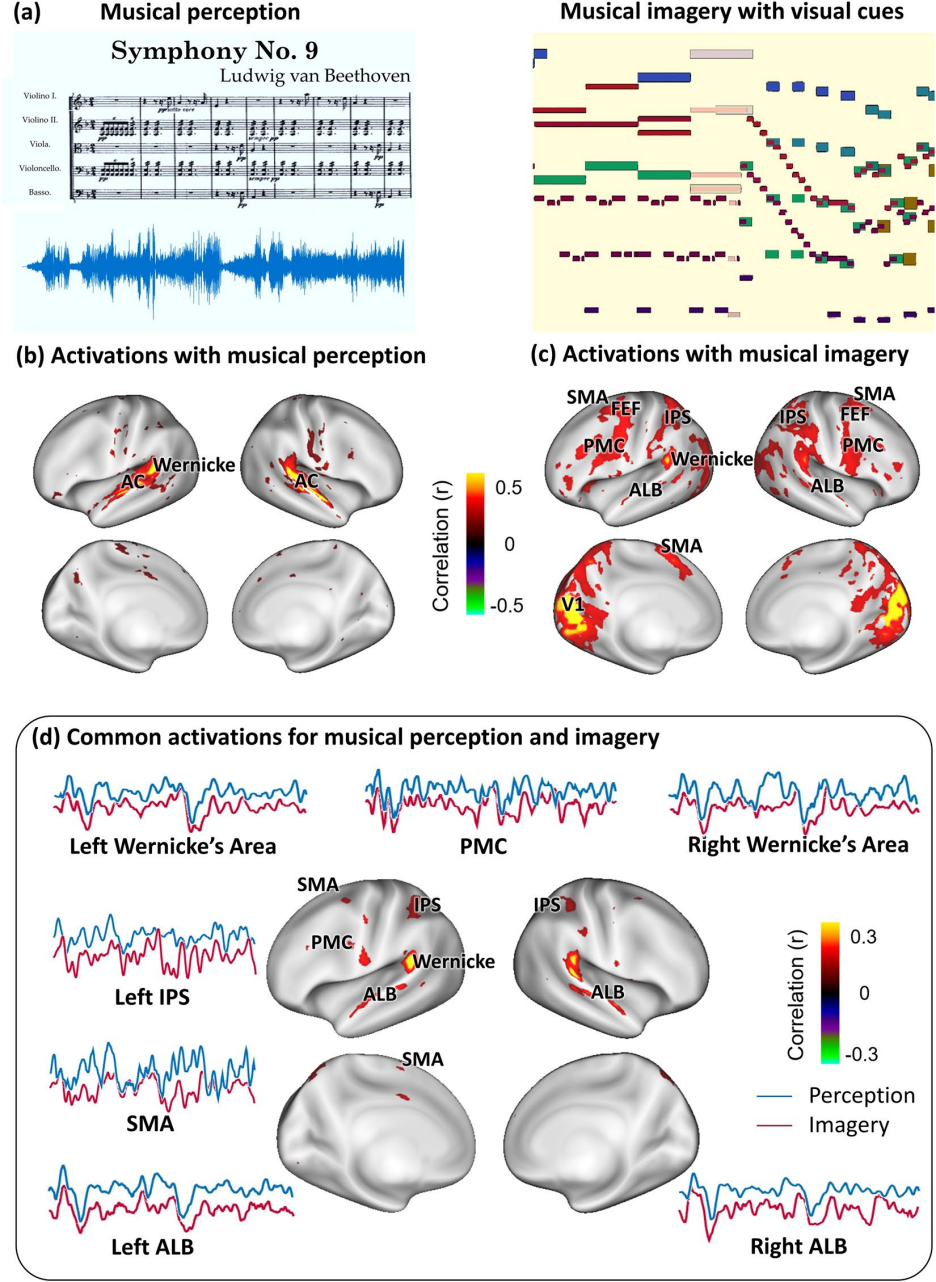
Distinct and common cortical activations with musical perception and imagery. (**a**) Paradigm for musical perception (left) and imagery (right). This music is in the public domain. The music score in (**a**) was downloaded from http://www.free-scores.com/download-sheet-music.php?pdf=1220#. The visualized music shown in (**b**) is an animation with bars moving from right to left as the music flows. It includes all the musical information as in a standard music sheet: the length of the bars indicates the note length (rhythm and duration); the height of the bars indicates the keynote (pitch); the color of the bars indicates the instrument (timbre). (**b**) Cortical activations for musical perception (two-tailed significance level p < 0.01). (**c**) Cortical activations for musical imagery (two-tailed significance level p < 0.005). (**d**) Shared cortical substrates between musical perception and musical imagery (two-tailed significance level p < 0.01). The time series was extracted from the fMRI signal averaged across the perception or imagery sessions from the labelled locations (AC: Auditory cortex; ALB: Auditory anterolateral belt; PMC: Premotor cortex; FEF: Frontal eye field; IPS: Intraparietal sulcus; SMA: Supplementary motor area).

When a subject with musical training listened to an 8-min music piece eight times during the fMRI acquisition (Fig. 1a, left), activated cortical areas were mapped by identifying the voxels with reproducible fMRI signals across repetitions. Activated voxels were mostly confined to the auditory cortex, including the core and belt regions along the ventral auditory pathway, and Wernicke’s area (Fig. 1b); the activations were slightly stronger in the right hemisphere than the left hemisphere. Whereas this was unsurprising given the auditory nature of the musical-perception task, we further asked how the brain engaged cortical processes to support musical imagery in the absence of any auditory input.

To address this question, we visualized the music as a silent movie (Fig. 1a, right). This movie provided real-time visual cues to inform subjects of the content of musical imagery and assisted in controlling the timing of the imagery process during the 8-min session. By watching this movie, each subject could consistently imagine the music piece for 12 repeated sessions of fMRI scans. Similar to the activation analysis for musical perception, the cortical activation during the visually-cued musical imagery was mapped by assessing the intra-subject reproducibility of fMRI signals at the voxel level. The activated areas covered a large part of the cortex, including the primary visual cortex, dorsal visual areas, the parietal association cortex, the anterolateral belt, Wernicke’s area, the frontal eye fields, the supplemental motor area and the premotor cortex (Fig. 1c). The responses at these areas could be attributable to either visual stimuli or musical imagery, since the task required the subject to process the visual cues as well as to imagine the music accordingly.

### Musical imagery and perception shared common cortical substrates

We further compared the task-evoked responses between the imagery and musical perception conditions. This allowed us to localize the responses to musical imagery as opposed to the visual stimuli because no visual stimuli were given during the perception condition. Since the anterolateral belt and Wernicke’s area were activated by both tasks, they were likely the shared cortical substrates for both musical perception and imagery. To test this hypothesis, we assessed the fMRI signal correlation between each musical-imagery session and each music-perception session at each voxel. This analysis revealed the areas that showed consistent responses to both tasks (Fig. 1d). Such areas included Wernicke’s area on the left hemisphere and its homologous area on the right hemisphere (herein we refer to them as the bilateral Wernicke’s areas), and to a lesser degree the anterolateral belt, the supplementary motor areas, and the premotor cortex. Note that the perception task involved only auditory input but not visual input, whereas the imagery task involved only visual input but not auditory input. The voxel-wise correlation across these two task conditions was only attributable to the common music content in both conditions, regardless of whether it was actually presented or mentally recreated. As such, Fig. 1d reports the cortical areas that underwent the same processing during sustained musical imagery and perception. By visual inspection of the averaged fMRI signals, we found that both the imagery and perception tasks evoked complex but similar responses bilaterally in Wernicke’s areas, supplementary motor areas, and premotor cortex, over the entire duration of the music stimulus (Fig. 1d).

In four additional sessions, we also presented the music with the same visual display as used in the musical-imagery task, yielding a control condition for us to validate findings from the intra-subject reproducibility analysis. As expected, the correlation in the fMRI signal between the (auditory-only) perception condition and the (auditory and visual) control condition revealed shared activations in the auditory cortex but not in the visual cortex (Supplementary Information, Fig. S1a). This was similar to the activations uncovered during auditory-only perception (Fig. 1b) as the subject listened to the same music in both conditions but only watched the visualization of the music in one condition. The correlation between the (visual-only) imagery condition and the (auditory and visual) control condition revealed shared activations at both the auditory and visual cortices (Supplementary Information Fig. S1b). The common activation in the visual cortex was expected because the visual stimuli drove reproducible responses at areas that processed the stimuli. However, the shared activations in Wernicke’s area and the anterolateral belt further confirmed that these two areas were involved in both musical perception and imagery, despite the presence or absence of the visual display in the musical-perception condition.

### Consistency and variation across subjects

**Figure 2:**
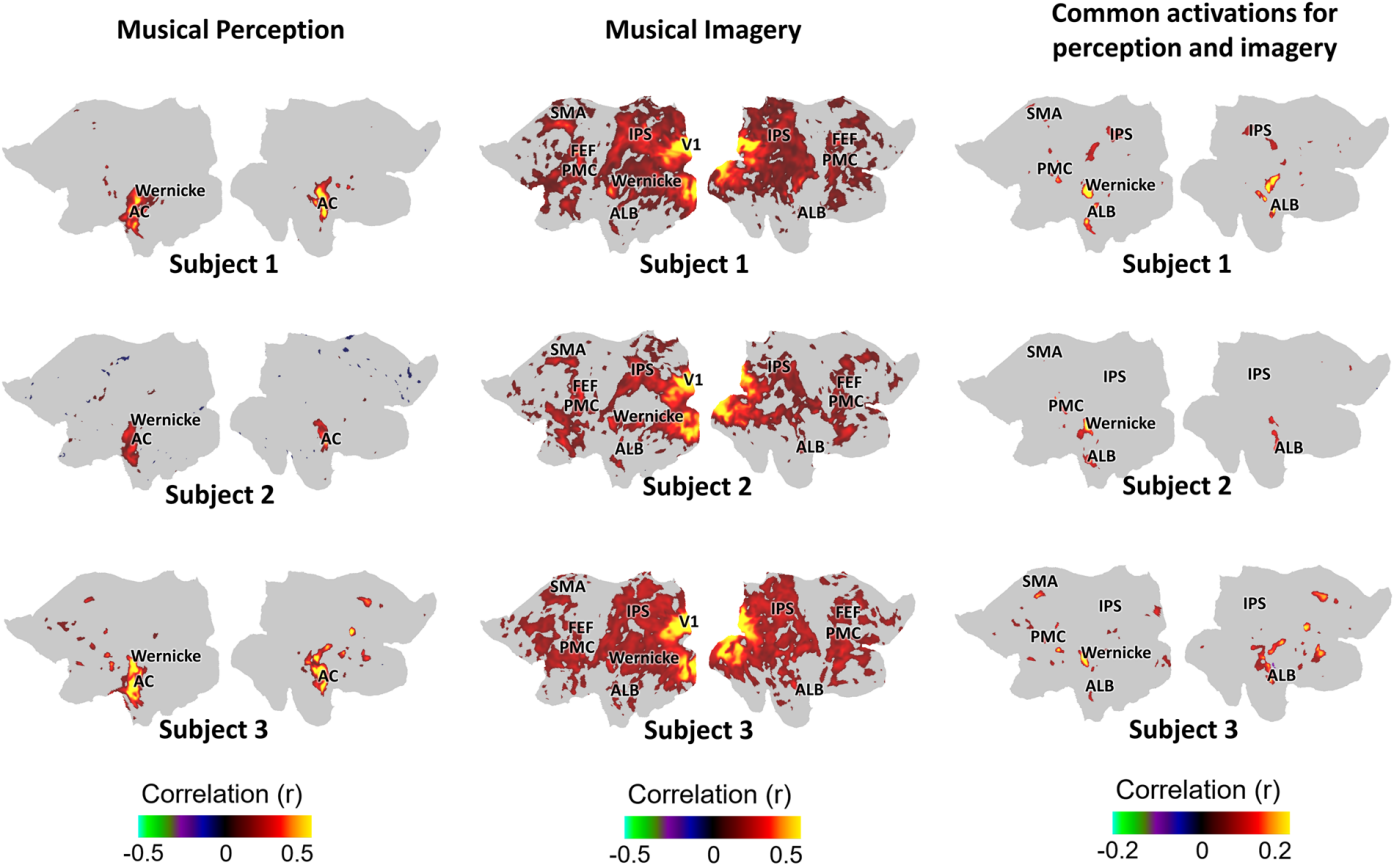
Cortical activations with musical perception and imagery were consistent across all subjects. Each row indicates the cortical mapping for one subject. The first column shows the cortical activations for musical perception; the second column illustrates the cortical activations for musical imagery; the third column shows the shared cortical substrates between the two tasks. The first row (subject 1) depicts a flattened view of surface mapping as Fig. 1. (**b**–**d**) (AC: Auditory cortex; ALB: Auditory anterolateral belt; PMC: Premotor cortex; FEF: Frontal eye field; IPS: Intraparietal sulcus; SMA: Supplementary motor area; V1: Primary visual cortex).

As shown in Fig. 2, the above findings observed in one subject were also reproduced in two other subjects with similar cultural and musical training backgrounds. In these subjects, the musical-imagery task was also repeated 12 times and the musical-perception task was repeated 8 times.

**Figure 3:**
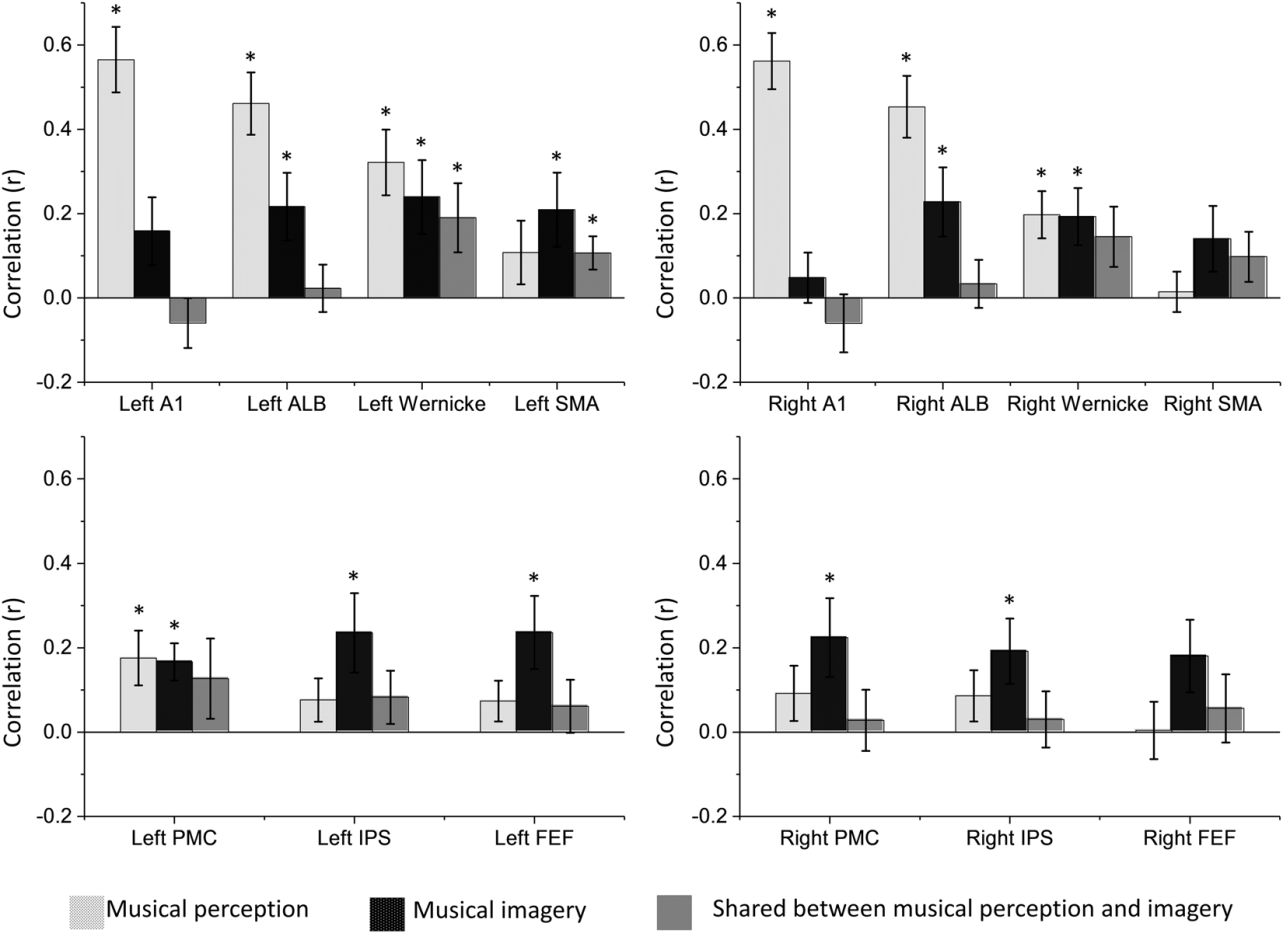
Distinct and common cortical activations with musical perception and imagery from group-level analysis. Each chart reflects the averaged correlation (r value) among all subjects in different regions of interest (ROI) compared across three conditions: reproducibility between musical perception sessions (light gray); reproducibility between musical imagery sessions (black); correlation between a musical perception session and a musical imagery session (dark gray). The left two charts show the results for ROIs in the left hemisphere and the right two charts show the results for ROIs in the right hemisphere. The mark * over a bar indicates that the specific ROI is consistently significantly activated by the musical perception or imagery task, or co-activated by both tasks among all subjects (two-tailed significance level p < 0.05). (A1: Primary auditory cortex; ALB: Auditory anterolateral belt; SMA: Supplementary motor area; PMC: Premotor cortex; FEF: Frontal eye field; IPS: Intraparietal sulcus).

Beyond single-subject analysis in these three subjects, we also generalized the findings to a larger group of eight subjects who underwent fewer sessions (two under musical perception and two under musical imagery). Although the smaller amount of repetitions reduced the statistical power and the signal to noise ratio in each subject, the intra-subject correlations within and between perception and imagery sessions based on a set of regions of interest (ROIs) were averaged across subjects to reveal the group-level effects. In general, the group-level results revealed a similar set of activated regions as those obtained at the single subject levels (Figs 1 and 2): the auditory core and belt areas, bilateral Wernicke’s areas, and the left premotor cortex were activated during musical perception; while bilateral Wernicke’s area, auditory anterolateral belts, premotor cortices, Intraparietal sulcus, left supplementary motor area and left frontal eye field were activated during musical imagery (Fig. 3). The overall activations in left hemisphere are more consistent across all subjects than those in the right hemisphere. Particularly, the correlation between musical perception and imagery was most pronounced in Wernicke’s area in the left hemisphere, suggesting that its role in musical imagery was most reliable and consistent across subjects.

### Responses at Wernicke’s areas coded music-timbre features during musical imagery

**Figure 4:**
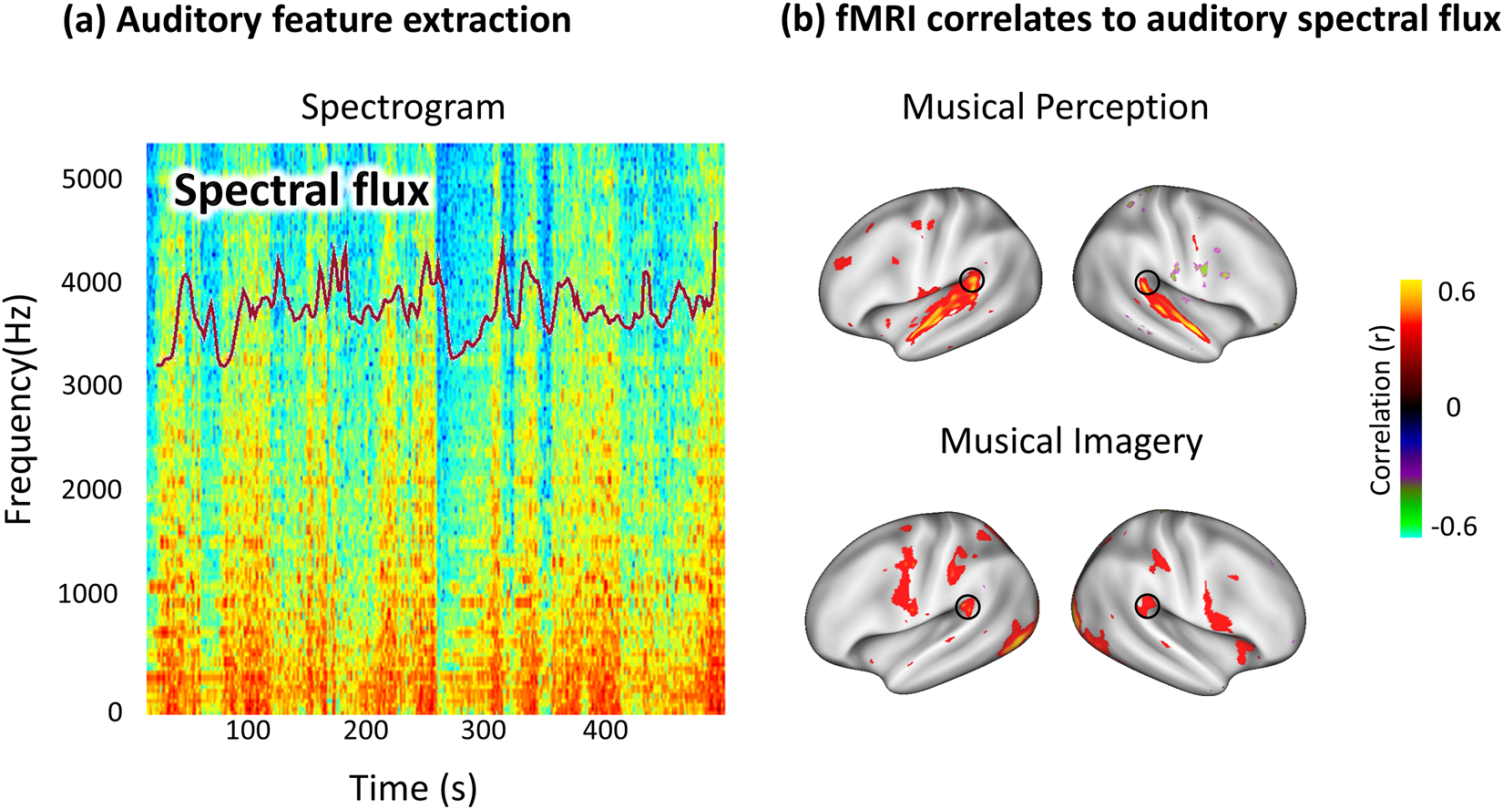
Responses at Wernicke’s areas coded musical features during imagery. (**a**) The auditory spectral flux was extracted from the stimulus spectrogram as a feature showing how quickly the power spectrum of a sound wave changes over time. (**b**) Spectral flux was highly correlated with the fMRI signals (averaged across all subjects) in the common cortical regions shared between musical perception and imagery (corrected at false discovery rate (FDR) q < 0.05), especially in ventral visual areas and bilaterally in Wernicke’s areas (as circled on the maps).

We further asked what musical information was preserved in the brain during musical imagery. To address this question, we explored a specific auditory feature known as the spectral flux. This feature measures how quickly the power spectrum of a sound wave changes over time^24^, and it reflects the music timbre^25^. As shown in Fig. 4a, extracting the spectral flux from the music stimulus resulted in a time series that represented a timbre-related auditory feature in the music. After convolving the spectral flux with the hemodynamic response function (HRF) to produce its assumed signature in the fMRI signal, this feature time series was correlated to the real fMRI signal at each voxel during musical imagery. A finding consistent across subjects was that the significantly correlated areas included ventral visual areas and Wernicke’s areas in both hemispheres (Fig. 4b, bottom). The former was not surprising since the visual cues changed according to the content of the music. The latter was interesting, because the Wernicke’s area was not directly involved in visual processing. The correlation between the activity at Wernicke’s area and the spectral-flux fluctuation implied that Wernicke’s area represented auditory information during musical imagery, despite the absence of acoustic stimuli. During musical perception, the spectral-flux fluctuation was also correlated with the activity in Wernicke’s areas, as well as in the core and belt areas in the auditory cortex (Fig. 4b, top). Nevertheless, Wernicke’s area was the only area that was correlated to the spectral-flux feature in both musical perception and musical imagery. Similar observations were obtained when a different musical feature was extracted from the amplitude envelope of the sound – another important feature for music timbre^26^ (see Supplementary Information Fig. 2). Taken together, these results suggest the auditory nature of neural coding in Wernicke’s area during both musical perception and imagery.

### Musical imagery and perception evoked highly distinctive cortical networks

**Figure 5:**
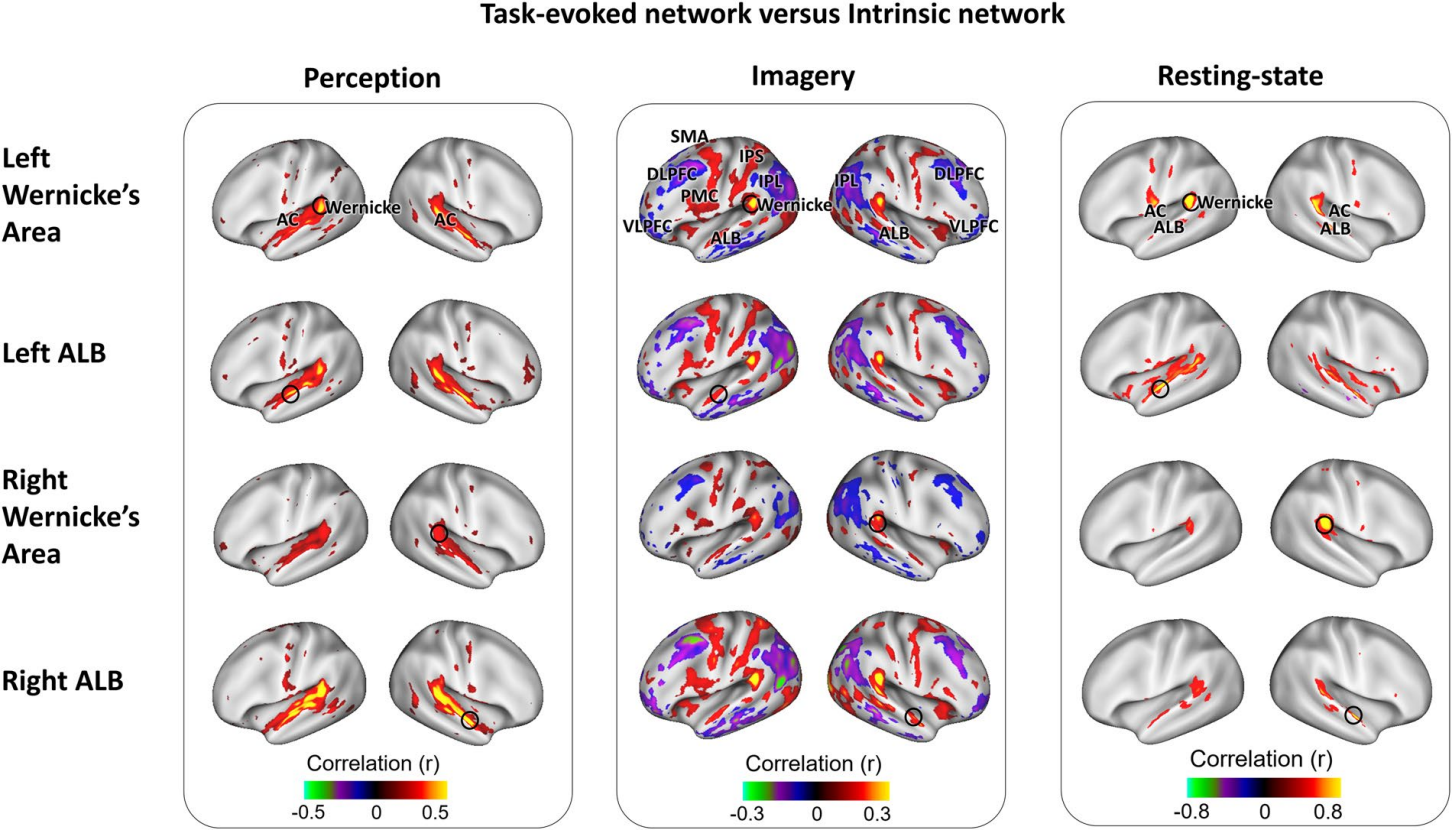
Task-evoked cortical network versus intrinsic network. The perception-evoked (first column) and imagery-evoked (second-column) cortical networks were mapped by cross-session seed-based correlation in the task-related fMRI signals. The third column is the intrinsic network mapped with resting-state fMRI. Each row corresponds to one specific seed location (circled on the maps), labelled on the left. (AC: Auditory cortex; ALB: Auditory anterolateral belt; PMC: Premotor cortex; IPS: Intraparietal sulcus; SMA: Supplementary motor area; IPL: Inferior Parietal Lobule; DLPFC: Dorsal Lateral Prefrontal Cortex; VLPFC: Ventral Lateral Prefrontal Cortex).

We also explored how the brain engaged its functional networks to support musical imagery and perception. For this purpose, we mapped and compared the task-evoked patterns of functional connectivity during the musical imagery versus during the music perception task. This analysis was based on seed-based inter-session functional connectivity^27^, which isolates task-evoked responses that are reproducible and correlated across different sessions of the same task^23^, becuase spontaneous activity is independent of the task and thus uncorrelated across sessions. In this regard, four seed locations were chosen bilaterally from Wernicke’s areas and anterolateral belts, because these areas were activated by both the musical imagery and perception tasks. Notably, the task-evoked patterns of seed-based functional connectivity were highly distinctive between the musical-perception task and the musical-imagery task (Fig. 5). In contrast to the unimodal network involved in musical perception (Fig. 5, left), musical imagery recruited a much broader and more complex pattern of network interactions (Fig. 5, middle). During musical imagery, the four seed locations were positively correlated with the attention network (including the intraparietal sulcus and the frontal eye fields) and the motor-control network in the prefrontal cortex (including the supplementary motor area and the premotor cortex), but negatively correlated with the default-mode network (including the posterior cingulate cortex, inferior parietal lobule, medial and lateral prefrontal cortices). Both the correlated and anti-correlated networks were largely symmetric between the two hemispheres. Although the network patterns appeared distinct between musical imagery and perception, the correlations between Wernicke’s areas and the anterolateral belt cortex were noticeable in both of the tasks.

We further asked whether the patterns of network interactions during musical imagery or perception reflected the intrinsic functional connectivity during the resting state in the absence of any task. The resting state networks were consistent with the networks evoked by musical perception (Fig. 5, right). However, the networks evoked by musical imagery were not confined to any single intrinsic network; instead they involved complex interactions of multiple networks, including higher-order auditory networks, the attention network, the motor-control network, and the default-mode network.

## Discussion

In this study, we introduced a new experimental paradigm to map and compare cortical activations and networks in musicians during sustained and complex musical imagery versus during musical perception. During visually-cued musical imagery, subjects watched a movie and accordingly imagined a specific music piece. Wernicke’s area and its homologous area in the right hemisphere facilitated the re-creation of the auditory features of the music, giving rise to vivid musical imagery resembling the experience of actually listening to music. Although Wernicke’s area was involved in both musical imagery and perception, its interactions with other brain regions appeared to be highly different between these two conditions: musical imagery elicited much more widespread, complex, and multi-modal networks, whereas musical perception recruited intrinsic and unimodal networks. The most notable network signature of musical imagery was the anti-correlation between the default-model network and task positive networks for attention or motor control.

### Music visualization facilitates repeated measures of sustained musical imagery

Mental imagery is a subjective human experience that is hard to control and measure. Previous studies have mostly utilized experimental paradigms to acquire short periods of musical imagery alternating with other conditions, e.g. musical perception and resting state^3,15,16^. Although such paradigms are convenient for mapping cortical activations with musical imagery, they do not allow repeated observations of brain activity during sustained musical imagery task such that patterns of task-evoked network interactions can be mapped.

Unique to this study is the use of music visualization to control the timing and inform the content of complex and sustained musical imagery. The visualization uses a similar concept as Karaoke, enabling subjects to imagine the same music with a controlled tempo. This allows for repeated measures of the same imagery experience within and across subjects, so that the fMRI scans can be compared across sessions to map the imagery-evoked activations and networks, as previously demonstrated in studies for natural vision or hearing^22^. Moreover, this stimulus paradigm also allows direct comparison of the fMRI signals between imagery and perception to map their shared cortical representations.

In addition to the purpose of mapping imagery-evoked activations and networks, this visually-cued imagery paradigm also offers a new opportunity to study neural coding for imagery. Many musical features can be predicted by decoding fMRI data from subjects listening to music^28,29^; however, it is very difficult to develop or evaluate methods for decoding musical imagery. In general, brain decoding methods require extensive data for training and testing decoding models^30,31^. Such data are currently unavailable for mental imagery because the content of imagery is unsynchronized, complex, and inaccessible. Thus, it is desirable to experimentally control the imagery content to allow for synchronized and repeated brain activity measurements to generate data for rigorously training and testing decoding models, before attempting to apply them to more complex forms of imagery or cognitive processes, such as dreams^32^.

### Musical imagery involves bilateral Wernicke’s areas

Our results suggest that two areas are essential to musical imagery: the anterolateral belt and Wernicke’s area. As a part of the ventral auditory pathway, the anterolateral belt is likely involved in the recognition of the sound information during musical imagery. However, it was unexpected that Wernicke’s areas were bilaterally activated with similar responses during musical imagery and perception (Fig. 1). Wernicke’s area is conventionally considered to be responsible for language processing and comprehension^33,34^. However, the stimulus in this study was a recording of classical music without lyrics, and did not involve language per se. Therefore, the response in Wernicke’s area was unlikely to reflect language processing during either musical imagery or processing. This finding led us to speculate the functional role of Wernicke’s area beyond language^34^.

In fact, music and language are both carried by sequential inputs with hierarchical syntactic structures^35^. The syntax in language refers to the principles of grouping words into phrase, and phrases into sentence. Likewise, the syntax in music refers to the principles of combining tones into chords, and chords into harmony^36^. Given their syntactic similarities, language and music processing might utilize a partly shared set of neural substrates^37–39^. Among them, Wernicke’s area is likely responsible for general syntax processing of sequences for language as well as music, as the information is represented through similarly hierarchical syntactic integration^40^.

It is conventionally thought that music is processed by the right hemisphere, whereas speech is processed by the left hemisphere^41^. However, such hemispheric specialization has been challenged by findings from recent functional imaging studies, which suggest involvement of both hemispheres in music perception^42,43^. Our results support and extend this notion by further demonstrating that both hemispheres are involved in not only musical perception but also in musical imagery. It also turns out that music processing activates the networks rather symmetrically in both hemispheres. Future studies are desirable to explore the potentially distinctive roles of the left and right hemispheres during musical imagery.

### Wernicke’s area encodes music timbre information during musical imagery

In this study, the response in Wernicke’s area was highly correlated with music timbre features of the stimulus as imagined or heard (Fig. 4 and Supplementary Information Fig. 2). This observation offers insights about the previously undiscovered function of Wernicke’s area. At least in musicians, Wernicke’s area may encode musical information, whether it is internally generated (imagery) or externally stimulated (perception), beyond its known function in language comprehension^33,44^.

The musical information represented by the activity of Wernicke’s area reflected low level features of the sound, such as spectral or amplitude fluctuations. During musical imagery, the sound was not present; therefore, the mentally-generated acoustic features were likely driven through top-down processes, as opposed to bottom-up processes that occurred when the sound was physically presented. In this context, the top-down and bottom-up processes involved distinct areas and networks. When the music was presented externally as a sound wave, its processing involved the primary auditory cortex and the ventral auditory pathway. In contrast, when the music was imagined in mind, the primary auditory cortex was not involved in representing the music (Fig. 4). This distinction suggests that the primary auditory cortex encodes the auditory features of a music only when it is perceived and not when it is imagined^5^.

### Musical imagery requires complex interactions among multiple intrinsic networks

Although Wernicke’s area encoded auditory features of music for both musical imagery and musical perception, its functional connectivity appeared largely different in these two conditions (Fig. 5). In the musical perception task, Wernicke’s area interacted with the auditory cortices bilaterally, showing a pattern of task-evoked functional connectivity consistent with the intrinsic functional network in the resting state. However, during musical imagery, Wernicke’s area engaged itself in a more complex pattern of functional connectivity, which included a subset of the auditory network, the attention network, and the default-mode network.

This finding is intuitively reasonable, as musical perception is a more natural and less demanding task than musical imagery. The high attention demand during musical imagery explains the positive interaction between Wernicke’s area and the attention network^45^. It also explains the negative interaction with the default-mode network, which tends to be deactivated by attention-demanding tasks^46^ and anti-correlated with task-positive networks at rest^47^. Moreover, the auditory network evoked by musical imagery did not cover the whole auditory cortex; it only included Wernicke’s area and the anterolateral belt, and excluded the primary auditory cortex. This musical-imagery evoked network was notably different from the intrinsic auditory network observed in the resting state and from the task-evoked auditory network during musical perception. The absence of the primary auditory cortex in musical-imagery evoked network is consistent with findings from majority of the previous studies^3,16^, but not Kraemer *et al*.^15^, though this topic remains under debate.

Moreover, our results demonstrate that task-evoked functional networks may differ from intrinsic functional networks in the resting state, despite a general correspondence between them^48^. A task-evoked network may segregate up an intrinsic network to involve a subset of regions, or it may recruit multiple intrinsic networks through network-network interactions. The degree to which a task-evoked network corresponds to its intrinsic mode may directly depend on how natural and frequent a type of tasks occur in realistic experiences (listening to music is more naturalistic than imagining music) or may inversely depend on the cognitive load of the task.

### Supplementary and premotor motor areas are involved in musical imagery

In this study, the supplementary motor areas and the premotor cortex were activated during musical imagery, confirming findings in previous studies^3,14,16,17^. Since the musical-imagery task did not involve any movement and all subjects reported no humming or other vocalizations, the activations of these motor-related areas were unlikely to be attributed to movements, but resulted from the mental imagery state. In line with this interpretation, the supplementary motor areas and the premotor cortex were activated with imagery, whereas the primary motor cortex was not. However, we did not explicitly monitor vocal or sub-vocal movement (e.g. with EMG recordings), and thus could not entirely exclude the possibility of all kinds of vocalization.

Sensory and motor experiences are often linked to one another^18^. When playing an instrument, a musician reads music scores and controls fine motor movements to produce sound; the sound provides feedback for the musician to refine motor control^49^. Such sensorimotor interactions might also occur during musical imagery, involving a motor-control circuit in the prefrontal cortex, including supplementary motor and premotor areas. The motor-control network activated with musical imagery (Fig. 1c) appears to be similar to the network activated with motor imagery^50^. Thus, it is plausible that motor imagery (subvocal silent singing of notes or imagery of finger movements during music performing) may occur during musical imagery in musicians, or even during non-musical auditory imagery^17^.

### Musicians versus non-musicians

Musical imagery is not unique to musicians, and it is a common human experience that may even occur involuntarily to most people^51^. However, research on musical imagery has often tested musicians and non-musicians in separate studies, since musical-imagery tasks for musicians may be too difficult for non-musicians to complete^52^. For example, musicians are able to mentally imagine the temporal reversal of familiar melodies^53^, which is hardly possible for non-musicians. Likewise, the imagery task based on music visualization is also very difficult for non-musicians to perform, limiting the scope of this study to musicians with a long history of musical training.

Musicians and non-musicians may process and understand musical information in distinctive ways. For musicians, “reading the visualized music” or “hearing the music” might involve similar cognitive processes as reading and processing the musical note – the “language” specialized to music as a result of their musical training and knowledge. Musicians may also generate vivid imagery during silent reading of music scores, namely “notational audiation”. Previous studies have shown that this specific type of musical imagery may trigger both auditory and motor imagery in musicians^54^. The motor imagery may come from the subvocal silent singing of notes, or the manual motor imagery of performing instruments. In this study, we used a similar paradigm as “notational audiation”, except that our visualization of music was more intuitive and better controlled in timing. All subjects in this study were musicians with a long history of musical training or practice. They all reported experiencing vivid musical imagery during experiments, and could imagine the music with accurate tempo and pitch in mind. Some even reported being able to imagine the instruments being played during mental imagery of the Beethoven symphony. The visually-cued imagery task in this study may also elicit the engagement of sensorimotor interactions and even motor imagery (as discussed in previous section).

Musicians often outperform non-musicians not only during musical imagery tasks^55,56^ but also during non-musical auditory imagery tasks^57^. Previous functional neuroimaging study found enlarged auditory cortical representation in musicians, and the enlargement of tonotopic maps was correlated with their ages of starting musical training^58^. Our results also suggest that both musical imagery and perception evoke bilateral activations and networks in musicians. Recent evidence suggests that this breakdown of lateralization is more likely to be observed in musicians^59^. Since all participants in this study were trained musicians, we could not contrast our observations in musicians against non-musicians. As a result, we speculate, but do not assert, any causal relationship between musical training and imagery ability.

## Methods and Materials

### Subjects and Stimuli

Nine healthy volunteers (Age 19–27, 3 females, all right-handed with normal hearing and on average 10.9 years of musical training; see Table 1 for subject details) participated in the study with informed written consent obtained from each subject according to a research protocol approved by the Institutional Review Board at Purdue University. All experiments were performed in accordance with relevant guidelines and regulations as described in this approved protocol. Here, we focused on musicians because the task of imagining a long piece of classic music (elaborated as below) was rather difficult for non-musicians. Otherwise, inclusion of data from any subjects who were unable to perform the musical-imagery task would complicate the study, of which the main goal was to map cortical activations and networks underlying musical imagery. For this reason, any findings from this study were specifically confined to musicians with a long-term musical training.

**Table 1:** Subject information. This table shows the information of all 9 subjects regarding their gender, age, handedness and musical training experience.

Subject #	Gender	Age	Handedness	Musical Training
1	Female	23	Right-handed	Violin, 11 years
2	Male	24	Right-handed	Percussion, 7 years
3	Male	26	Right-handed	Piano, 10 years
4	Male	23	Right-handed	Tuba, 12 years
5	Male	23	Right-handed	Trombone, 16 years
6	Female	27	Right-handed	Piano, 12 years
7	Male	19	Right-handed	Piano/Trumpet, 13 years
8	Female	21	Right-handed	Flute/Bassoon/Piano, 12 years
9	Male	20	Right-handed	Piano, 5 years

As illustrated in Fig. 1a (left), the auditory stimulus was the first 8 minutes of the first movement of Beethoven’s Symphony 9 (sampling rate: 11025 Hz), delivered through binaural MR-compatible headphones (Silent Scan Audio Systems, Avotec, Stuart, FL). This music piece was visualized as a movie through Stephen Malinowski’s Music Animation Machine^20^. The visualization is a graphic music score based on the design of a 2-dimension piano roll. As illustrated in Fig. 1a (right), it is an animation with rectangular bars moving from right to left as the music flows; the current note is the highlighted bar in the middle of the screen. Each subject imagined the music while watching the movie visualizing music. The movie offered real-time visual cues to control the timing and inform the content of musical imagery. The visualized music was delivered through a binocular goggle system (NordicNeuroLab Visual System, Bergen, Norway) using Psychophysics Toolbox 3 (http://psychtoolbox.org).

Prior to fMRI, each subject was trained for 1 to 2 hours, to become familiar with the music visualization and imagery task. For the purposes of this study, 1 to 2 hours of training was sufficient, as the visualization was intuitive for musicians^60^. The subjects were instructed to report the vividness of their musical imagery based on a 1–5 scale (1 not at all; 5 very accurate). All subjects were able to vividly imagine the music, and self-reported 4.1 ± 0.6 for the accuracy of pitches, 4.7 ± 0.5 for the accuracy of tempo and 3.8 ± 0.9 for the accuracy of distinguishing different instruments. However, it should be noted that the above self-reporting by itself only provided subjective assessment of musical imagery, and could not be taken as fully objective evidence for the existence and vividness of musical imagery. Despite its limitation, the self-reported scores still offered reasonable support for the presence of musical imagery. Also note that the self-reported scores were not used in the fMRI analysis. So, the (subjective) behavioral assessment and the imaging-based evidence were entirely independent of each other.

### Experiment

In the musical perception sessions, each subject was instructed to listen to this music with his or her eyes closed while no movie was presented. During the musical imagery sessions, each subject was instructed to imagine the music piece while watching the silent music visualization. Three subjects performed the musical-perception task eight times and the musical-imagery task twelve times. The perception and imagery session order was counterbalanced but not randomized across subjects. The sessions were conducted over a period of five days; two perception sessions and two imagery sessions in each of the first four days; four imagery sessions in the fifth day. The purpose of the high number of repetitions was to reliably examine the cortical activations and networks evoked by each task separately and shared by both tasks at the individual-subject level. This was especially desirable for the imagery task, which was cognitively demanding and likely involved complex cortical processes that were more variable across subjects than the perception task. The other six subjects underwent two repetitions of the perception task intermixed with two repetitions of the imagery tasks in a single day. Among these six subjects, one subject was excluded due to excessive head motion during fMRI scans. Despite the limited repetitions of the latter subjects, data from these subjects, as well as from two sessions of data arbitrarily chosen from the first three subjects, served the purpose of mapping cortical activations and networks during musical perception and imagery at the group level.

Each of the first three subjects also underwent six sessions of resting-state fMRI on a different day. In each session, the subjects were instructed to rest for 8 minutes with the eyes closed, without falling into sleep. Since the resting-state sessions were long separated (by days) from the tasks of musical perception or imagery, it was unlikely that the subjects were conditioned to “play the music in their heads” with a similar temporal precision without any instruction by the researchers.

### MRI Acquisition and Preprocessing

MRI data were acquired in a 3 T MRI system (Signa HDx, General Electric Healthcare, Milwaukee) with a 16-channel receive-only phase-array surface coil (NOVA Medical, Wilmington). Structural MRI with T1 and T2-weighted contrast were both acquired with 1 mm isotropic resolution. Functional MRI data were acquired using a single-shot, gradient-recalled echo-planar imaging sequence with 3.5 mm isotropic spatial resolution and 2 second temporal resolution (38 interleaved axial slices with 3.5 mm thickness and 3.5 × 3.5 mm^2^ in-plane resolution, TR/TE = 2000/35ms, flip angle = 78°, field of view = 22 × 22 cm^2^). Each subject was scanned with fMRI for 26 sessions: 8 sessions during the musical perception task, 12 under the visually-cued musical imagery task, and 6 in the wakeful eyes-closed resting state. Each session was 8 minutes in length.

MRI/fMRI images were preprocessed using a similar pipeline as in the Human Connectome Project^61^. Briefly, all fMRI images were corrected for slice timing and motion, aligned to structural images, normalized to the Montreal Neurological Institute (MNI) space, transformed onto individual cortical surfaces, and then co-registered based on myelin density and cortical folding patterns. Following this step, slow trends in the fMRI time series were corrected by regressing out a fourth-order polynomial function. Then fMRI data were temporally standardized (with a zero mean and unitary variance) and spatially smoothed by applying a 2-D Gaussian kernel of 2 mm full width at half maximum.

### Mapping cortical activations during musical perception or imagery

For the three subjects who underwent the greater number of repetitions, we mapped cortical activations during musical perception and imagery. For each task, intra-subject reproducibility was calculated at voxel-level, as previously used for measuring the reliability of cortical activity during naturalistic stimulation elsewhere^22,23^. This reproducibility was measured separately for each voxel as the temporal correlation between different sessions of the same task: 28 inter-session pairs among 8 sessions for the perception task and 66 inter-session pairs among 12 sessions for the imagery task. To handle the dependences among the different inter-session pairs for assessing statistical significance test, we applied a parametric statistical test to the intra-subject reproducibility based on linear mixed-effects (LME) modeling^62^ implemented in AFNI (http://afni.nimh.nih.gov). Specifically, the Fisher-transformed inter-session correlation *z*_*ij*_ is expressed in a crossed random-effects model,

1 $${z}_{ij}={b}_{0}+{\theta }_{i}+{\theta }_{j}+{\varepsilon }_{ij},$$

where *b*_0_ is the effect of interest, the average correlation across all inter-session pairs, *θ*_*i*_ and *θ*_*j*_ are the random effects associated with the *i*th and *j*th sessions, respectively, *ε*_*ij*_ is the residual term, *i, j* = 1, 2, …, 8 (for perception), and i, *j* = 1, 2, …, 12 (for imagery). Using this approach, we localized the brain areas significantly activated during musical perception (p < 0.01) and musical imagery (p < 0.005). See more details about the LME in a recent paper by Chen *et al*.^62^.

To test this effect at the group level, we used data from the group of eight subjects, for whom each underwent two sessions of musical imagery and two sessions of musical perception from each subject. We defined regions of interest (ROI) based on the multi-modal parcellation of human cerebral cortex^63^, namely primary auditory cortex (A1), auditory anterolateral belt (ALB), Wernicke’s area, supplementary motor area(SMA), premotor cortex (PMC), frontal eye field (FEF), intraparietal sulcus (IPS) (see more details regarding the size and center location of each ROI in Table 2). For each subject, the fMRI signals from the same session were first averaged within every ROI. Intra-subject reproducibility was evaluated for each ROI by correlating the averaged fMRI signals between repeated sessions of the same task and then converted to z scores. Then, the reproducibility was averaged across subjects, and evaluated for statistical significance using a one-sample t-test (p < 0.05). This analysis was done separately for the musical-imagery task and the musical-perception task.

**Table 2:** ROI information. This table shows the information including the name, size and center location of each ROI for group-level analyses (A1: Primary auditory cortex; ALB: Auditory anterolateral belt; SMA: Supplementary motor area; PMC: Premotor cortex; FEF: Frontal eye field; IPS: Intraparietal sulcus). Size refers to the number of voxels within each ROI. The vertex index of the center voxel for each ROI is extracted from the standard 32k vertex surface meshes of Human Connectome Project^61^. We also used the anatomical xyz-coordinates (mm) in the MNI space to indicate the location of the center voxel in each ROI.

ROI name	ROI size (# voxels)	Center vertex index	Center anatomical coordinates (mm)		
			x	y	z
Left Wernicke	158	9740	−47	−36	4
Left ALB	144	31695	−60	−2	−15
Left A1	216	31410	−38	−24	−3
Left SMA	206	4891	−11	2	39
Left FEF	150	5771	−34	−4	26
Left PMC	367	19550	−56	4	1
Left IPS	539	14629	−32	50	30
Right Wernicke	176	9636	48	−28	8
Right ALB	100	31653	56	10	−11
Right A1	167	31629	42	−16	−3
Right SMA	190	1876	4	8	38
Right FEF	179	5093	31	0	27
Right PMC	252	18029	58	9	21
Right IPS	353	14317	26	−40	183

### Mapping the common activations between musical perception and imagery

For each subject, we also mapped the cortical regions where the musical perception and imagery tasks evoked the same response. For this purpose, we calculated for each voxel’s the temporal correlation between each perception session and each imagery session. As such, the cross-task correlation was measured for a total of 96 pairs of sessions given 8 perception sessions and 12 imagery sessions. The significance of cross-task correlation was evaluated for each subject, based on a crossed random-effects LME model^62^,

2 $${z}_{ij}={b}_{0}+{\kappa }_{i}+{\lambda }_{j}+{\varepsilon }_{ij},$$

where *κ*_*i*_ and *λ*_*j*_ are the random effects are associated with the *i*th session of perception and the *j*th session of imagery, respectively, *i* = 1, 2, …, 8, and *j* = 1, 2, …, 12. From this approach, we located the brain areas in which activities were significantly shared between musical perception and musical imagery (p < 0.01).

We also performed the ROI-based group-level analysis on the eight additional subjects; for each subject,  data were available from two sessions of musical imagery and two sessions of musical perception. Using the method described in the previous sub-section, we evaluated the intra-subject correlation between musical imagery and perception for each ROI, converted the correlation coefficients to z scores and then averaged them across subjects. The statistical significance for the group-averaged effect was tested using a one-sample t-test (p < 0.05).

For one subject, we also mapped the cortical regions in which the (auditory-only) musical perception and (visual display only) imagery tasks evoked the similar responses during the control condition (perception with visual display). For this purpose, we calculated the temporal correlation in the voxel time series between 1) each perception and each control session at each voxel and 2) each imagery session and each control session at each voxel, in a similar manner as how the mapping of the common activations between musical perception and imagery was performed. The significance of the cross-task correlation was evaluated for each subject using the crossed random-effects LME model^62^ as Eq. (2).

### Mapping the fMRI correlates to auditory feature time series

We further explored the musical content coded in the fMRI response. For this purpose, the spectral flux and amplitude envelope of the music stimulus (in the auditory form) was extracted by the MIRtoolbox in Matlab^64^. Both spectral flux and amplitude envelope are important sound features for music timbre. Spectral flux measures the change in the power spectrum of a signal, which is calculated as the 2-norm (also known as the Euclidean distance) between the normalized spectra from adjacent frames^24^. The envelope shows the global outer shape of the signal, which can detect musical events such as notes. The feature frame size was set to 1 second with a step size of 0.1 second. The auditory feature time series were convolved with the canonical hemodynamic response function (HRF)^65^. The resulting time series were correlated with the fMRI signal at every voxel. For both the musical imagery and perception tasks, the correlation was calculated after the voxel time series was averaged across all the repeated sessions of the same task. A one-sample t test was applied to localize the brain areas that were significantly correlated with the auditory features during perception (corrected at false discovery rate (FDR) q < 0.05) and imagery (corrected at false discovery rate (FDR) q < 0.05).

### Mapping task-evoked networks during musical perception and imagery

For the three subjects in which a higher number repetitions were conducted, we mapped the task-evoked patterns of functional connectivity during both the musical perception and the imagery task using a seed-based inter-session correlation analysis^27^. Specifically, the time series at a particular seed location was taken from one session; then, the seed time series was correlated with every voxel time series during a different session of the same task. Four seed locations were chosen: 1) in the left Wernicke’s area, 2) in the right Wernicke’s area, 3) in the left Auditory anterolateral belt, and 4) in the right Auditory anterolateral belt. These choices were based on activated regions for both musical perception and imagery, as highlighted in our results (Fig. 1). The inter-session functional connectivity was evaluated for each pair of distinct sessions of the same task. The significance of inter-session functional connectivity was also evaluated using the LME method^62^. See Eq. (1).

For each subject, we further compared the task-evoked networks with intrinsic functional networks observed with resting-state fMRI. Seed-based correlations of the spontaneous activity were mapped in the same seed locations as mentioned above. Briefly, the voxel time series were concatenated across the six resting state sessions for each subject, and temporal correlations were calculated using the concatenated data. The statistical significance was evaluated using two-tailed one-sample t-tests (corrected at false discovery rate (FDR) q < 0.01).

### Data availability

The datasets generated under the current study are available from the corresponding author upon reasonable request. The minimally preprocessed MRI/fMRI data are also available on https://engineering.purdue.edu/libi/lab/Resource.html.

## Electronic supplementary material

Supplementary Figures
