# Supplementary material for: Musical Imagery Involves Wernicke’s Area in Bilateral and Anti-Correlated Network Interactions in Musicians
Source: Sci Rep. 2017 Dec 6;7:17066. doi: 10.1038/s41598-017-17178-4 (PMC5719057; doi:10.1038/s41598-017-17178-4)
Supplement: Supplementary file 1 — Supplementary Figures [file 41598_2017_17178_MOESM1_ESM.pdf]

Supplementary Information for

Musical Imagery Involves Wernicke's Area in Bilateral and Anti-Correlated Network Interactions in Musicians

Yizhen Zhang[2,3], Gang Chen[4], Haiguang Wen[2,3], Kun-Han Lu[2,3], Zhongming Liu[*1,2,3]

[1]Weldon School of Biomedical Engineering, Purdue University, West Lafayette, IN, USA

[2]School of Electrical and Computer Engineering, Purdue University, West Lafayette, IN, USA

[3]Purdue Institute for Integrative Neuroscience, Purdue University, West Lafayette, IN, USA

[4]Scientific and Statistical Computing Core, National Institute of Mental Health, National Institutes of Health, Bethesda, MD, USA

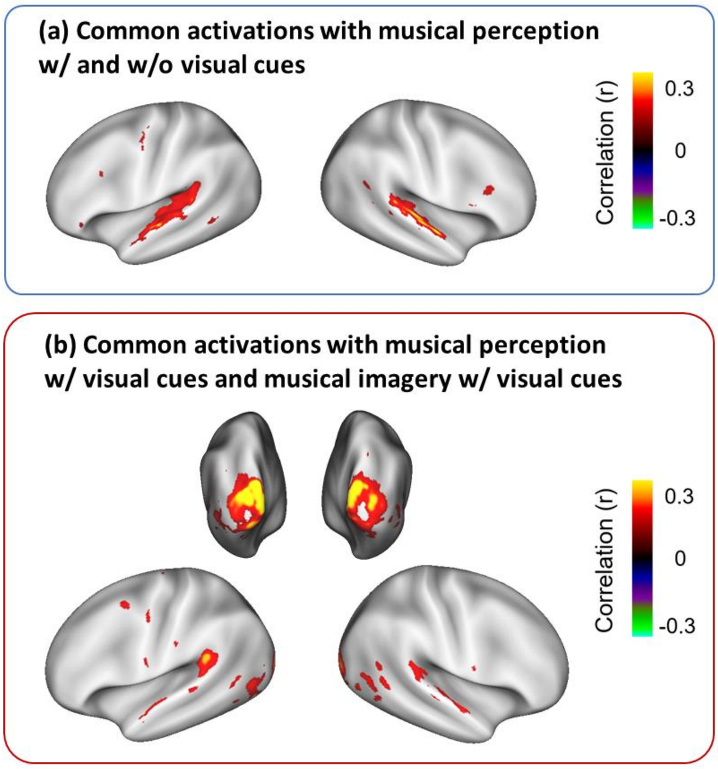

FigureS 1. Shared cortical activations between musical perception/imagery condition with control condition. (a) Cortical activations shared between the (auditory-only) perception condition and the control auditory-and-visual perception condition (two-tailed significance level p<0.05). (b) Cortical activations shared between imagery condition and the control auditory-and-visual perception condition (two-tailed significance level p<0.05).

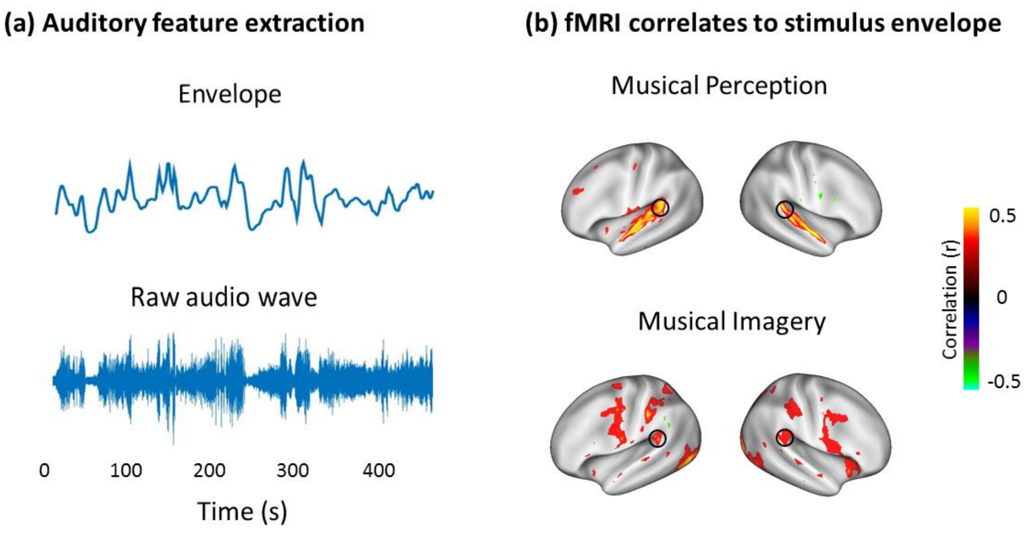

Figure S2. Responses at Wernicke's areas coded musical features (envelope) during imagery. (a) The amplitude envelope was extracted from the stimulus as a feature showing the global outer shape of the sound wave. (b) The amplitude envelope was highly correlated with the fMRI signals averaged among all subjects in the common cortical regions shared between musical perception and imagery (corrected at false discovery rate (FDR) $q<0.05$), especially in early visual areas and bilaterally in Wernicke's areas (as circled on the maps).
